# Supplementary material for: Predicting the effects of COVID-19 related interventions in urban settings by combining activity-based modelling, agent-based simulation, and mobile phone data
Source: PLoS One. 2021 Oct 28;16(10):e0259037. doi: 10.1371/journal.pone.0259037 (PMC8553173; doi:10.1371/journal.pone.0259037)
Supplement: S1 Appendix — (PDF) [file pone.0259037.s001.pdf]

S1 Appendix

Müller et al.

October 15, 2021

1 Details of the mobility model

Handling of large facilities The resolution of our input data comes at the level of "facilities". Those can be interpreted as buildings or sometimes blocks. They often contain multiple households, multiple company offices, multiple leisure facilities, multiple shops, etc. For *home* activities, we split persons living in the same facility into realistic household sizes with a maximum number of six people per household [1]. This seems important since the within-household dynamics of COVID-19, and in particular the fact that the secondary attack rate in households seems to be far below 100%, plays an important role (e.g. [2]). For all other activities, we divide the facilities by some globally set factor, called $N^{spacesPerFacility}$. That is, if two persons spend overlapping time at the same facility, the probability that they have interacted is $1/N^{spacesPerFacility}$. This has important ramifications for multi-day modelling and mixing, see below.

$N^{spacesPerFacility}$ evidently influences the number of contacts that a person has. For our simulations, we set it such that that number of contacts is roughly consistent with real-world contact tracing. For our current input data, that leads to a setting of $N^{spacesPerFacility} = 20$.

Multi-day modelling Optimally, one would have multi-day trajectories. In our case, the data that we have ends at the end of the day. Our simulations thus run the same person trajectories again and again (except for weekends, see below). This presumably *underestimates* mixing, since it is plausible to assume that there is some variation in activity patterns from day to day. At this point, one needs to make a decision whether our sub-spaces (see above) are frozen, meaning that the same sub-groups meet every day, or not. Using the same sub-groups every day arguably is plausible for office buildings, which may contain offices for several companies, and interaction may be limited to sharing an elevator. It is less plausible for public transport trains, where passengers are arranged differently every day. Possibly, a mix between the two approaches is plausible, introducing the need for even more free parameters. In our present model, we opt for the non-frozen setting, i.e. the other persons within a facility that an ego person interacts with are randomly re-drawn for every new simulated day.

Weekend modelling As already alluded to above, we use separate models for Saturdays and Sundays. They come out of transport modelling in the same way as we obtain the model for a "typical weekday" (see above). These models use the same synthetic persons and facilities, and thus can be aligned with the weekday model. In consequence, each synthetic person in our models, starting on Monday, (a) repeats the same weekday five times, (b) runs her Saturday schedule, (c) runs her Sunday schedule, and then starts over.

25% sample For computational reasons, we use a 25% sample of the full population. The sample is constructed by choosing 25% of all persons in the population randomly and retaining their full trajectories. The splitting of households as described above is done *after* the sampling, meaning that we have realistic household sizes in the 25% scenario but consider only 25% of them; also, the number of contacts to determine the parameter $N^{spacesPerFacility}$ (see above) is determined for the 25% model. We have also run the full 100% model to check that there are no major

differences. The 25% model allows to finish runs within a single-digit number of hours, which was and is important for fast model turn-around driven by the the necessity for fast progress given the demand for the results by the decisionmakers. All results are reported after upscaling to 100%.

2 Case numbers and hospital numbers in Berlin

The simulation is calibrated against the Berlin case numbers and the Berlin hospital numbers. COVID-19 is a notifiable disease, and the notifications are collected and published by the Robert Koch Institute (RKI) [3]. Each record contains at least two dates: The date when the record reaches the local health department (reporting date), and the date when symptoms started, called reference date.

In principle, the reference date would be easier to compare with our simulations, since it corresponds to the onset of our *showingSymptoms* state. Unfortunately, however, it is not clear how reliable that date is. The health department becomes aware of cases once they are tested positive. The positive test result becomes available about 2 days after the probe was taken. The health authorities thus have to connect a positive test with the person, and query the person about when symptoms started. Self-reported dates of symptoms onset are presumably rather unreliable, in part because of recall errors, in part because what a symptom is is not sharply defined. In addition, when tests are taken from pre- or asymptomatic cases, a date of symptoms onset is not yet available, and for asymptomatic cases never will be. In such cases, the reporting date is also entered as reference date, which for pre-symptomatic cases is too early. Finally, many records are reported completely without this reference date. RKI provides a procedure to impute the missing reference date [4], but has to rely on the statistical distribution of the cases where a reference date exists, which may not be a valid assumption since, say, locations that are under stress of high infection numbers may both not enter the reference date *and* receive the test results with additional delay. Also, the sampling strategy for testing was changed several times.

In consequence, we plot the case numbers both by reporting and by reference date for comparison, and also add a third number: The fraction of positive tests. In a targeted testing regime, this fraction will go up when testing is made more restrictive, and the other way around. It will thus react to changes in the testing regime in the opposite direction as the case numbers. In practical terms, we normalize the fraction curve such that it coincides with the cases curve in autumn, and is above the cases curve during all other times. This leads to a plausible corridor, i.e. between the reported case numbers (too low) and the rescaled test rate (too high) for the time series of the newly symptomatic in the simulations.

Because of these issues, we mostly calibrate against the hospital numbers in Berlin [5]. We believe those to be relatively unbiased, since there was always sufficient hospital capacity in Berlin throughout the period considered here.

3 Masks, contact tracing, and summer disease import

Masks In April the wearing of masks in shops and in public transport vehicles became obligatory in Berlin [6]. We have included this into the infection model of Eq. 1 of the main paper by reducing *sh* (if the contagious person wears a mask) and *in* (if the person to be potentially infected wears a mask). This is dependent on the activity type, meaning that persons only wear masks when shopping, doing errands or using public transport. Concerning the effectiveness of different mask types see Masks in the main paper.

The local transport company in Berlin (BVG, [7]) has provided us with the compliance rates in public transport over time meaning that we do not have to estimate them (cf. Fig 1). We assume that the same compliance rates also apply to shopping activities. We assume that 90% of those people wearing masks wear cloth masks and 10% wear N95 masks.

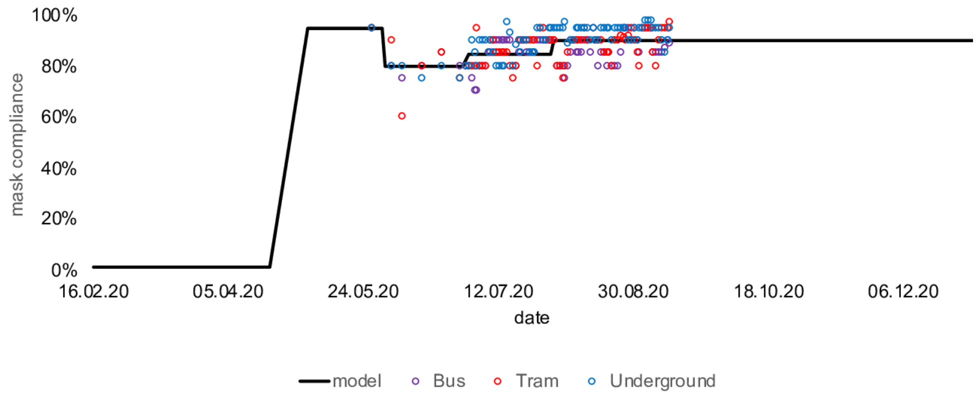

Figure 1: Mask compliance rates over time. Data provided by BVG [7].

Test, trace, and isolate (TTI) The goal of contact tracing is to break chains of transmission by tracing the contacts of an infected person and putting these contacts into quarantine. In our model contacts are traced during all activities except for public transport and shopping because we assume that the health authorities are not able to find these contacts. A contact person is only traced when the contact duration is longer than 15 minutes, which corresponds to the RKI guidelines [8].

Persons that go into *showingSymptoms* are assumed to trigger a contact tracing mechanism, which works as follows:

1. Look at all traced contacts that the infected person had in the 2 days [8] before showing symptoms.
2. A probability γ determines if a contact person can be reached successfully and also follows the stay-at-home order. γ is set to 0.5
3. The persons that have been traced successfully go into quarantine, but only after a delay of d days, which allows to model the response time of the system. Our base value of d is set to 5 days. Personal experience in our surroundings says that tests are normally taken a day after symptoms start, and the result is available again one day later in the evening. That is, contact tracing can start no earlier than 3 days after symptoms onset. We add another two days to account for possible additional delays.
4. A tracing capacity limits the number of persons per day for which its contacts can be traced. The capacity is set to 0 until the end of March, 40 cases per day until 14/Jun, and to 200 cases per day afterwards. Germany had agreed on a limit of 50 cases per 100 000 inhabitants per week at which local governments were expected to act [9]. This number was based on what the system presumably could handle for contact tracing. For our Berlin scenario with 5 million persons, this translates to 357 cases per day. Based on newspaper reports [10], the system was overwhelmed already at lower numbers, which is why we use 200.
5. Persons leave the home quarantine after 14 days, if they did not develop symptoms during that time.

For d, a smaller value would be much better in terms of effectiveness, but our personal experience in several cases says that this is unrealistic. For γ and the maximum tracing capacity, we compared simulation results.[1] Changes in γ make relatively little difference. For the maximum tracing capacity, one can see that larger capacities would have kept the new infections under control for longer than what happened in reality.

Combined effect of masks and contact tracing Masks and contact tracing do not have a strong enough effect to gauge them from the infection or hospital numbers. As explained in Reproduction number per activity type in the main paper, masks in public transport and while shopping reduces R by about 0.1 each. Since masks were introduced in April, they reduce the slopes of all curves of Fig 11 of the main paper accordingly. This makes the blue curve from that figure

[1] `https://covid-sim.info/2020-11-09/tracing`

(which is without masks) less plausible and the orange curve more plausible, which is welcome since the larger thetaFactor is more plausible (cf. Unrestricted model in the main paper).

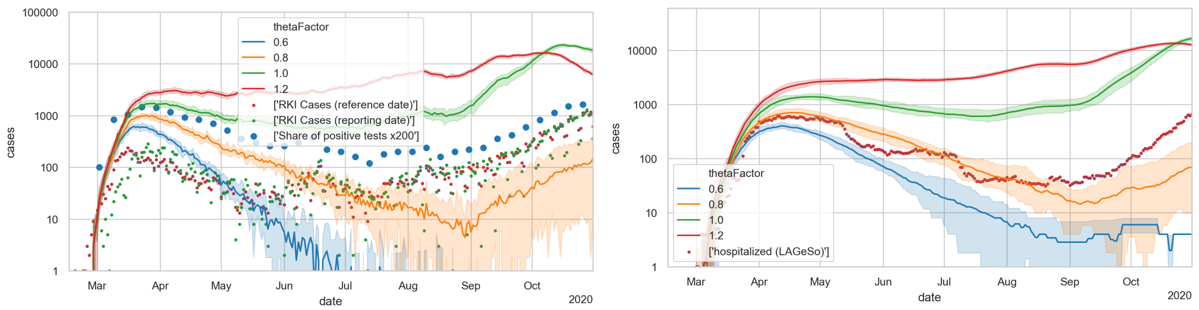

Figure 2: Case numbers (LEFT) and hospital numbers (RIGHT) in simulations that now also include masks and TTI (trace, test, and isolate).

Contact tracing, in contrast, just pulls the infection numbers down while they are low. Once contact tracing is overrun, it no longer influences exponential growth, and thus not the slopes of the second wave in the logplot.

We leave both of these elements in the model, since they are plausible by itself, their functioning is derived from first principles, and they have beneficial consequences. As stated, just based on the data alone, the case to include them would not be strong enough. The result can be seen in Fig 2.

Summer disease import After adding masks and contact tracing, the second wave is once more too late. Adding summer disease import pushes the curve up again (Fig 3). Other than during the spring import, where we multiplied the RKI numbers by 4, we now divide them by two. The reason is that the disease import stems from the case numbers, and as can be seen in the figure, the factor between the case numbers and the re-scaled positive test fraction is much smaller in summer than in spring (also cf. Under-reporting, and its variation over time in the main paper). Setting the disease import to one, as would be plausible by this argument, leads to an influence that is too large. Arguments why factor of one is too large are:

- Since there was widespread screening and an obligation to remain into quarantine-at-home for many people returning from summer travel, we argue that (1) the testing-and-quarantine regime had the consequence that many disease imports did not reinfect others and that (2) the screening also found many asymptomatic cases that would otherwise not have been included into the case numbers.
- In spring 2020, people travelled from an area without infections into an area with infections, and then came back, possibly infected. Had they stayed at home, they would not have become infected. In summer 2020, the situation was different: Had they stayed at home, they could have become infected as well. In consequence, only returns from areas with larger incidence then Germany would count as true disease import. Thus, most of the numbers labelled "probable infection abroad" by RKI cannot be counted as true disease import.

Again, the case for adding this element, and in this way, is not very strong. An alternative would have been to stay with the model of Fig 11 of the main paper, with a thetaFactor between 0.6 and 0.8 (look in particular at the hospital cases). Again, we prefer adding masks, contact tracing, and summer disease import, since the models can be constructed from first principles, and as a package, these three elements allow for a slightly larger Θ, which overall seems plausible.

4 Selection of geographical region and computing times

The project originally started with a scenario of the Berlin region, since we had an open scenario available [11] and thus did not have to wait for the data. With the data for all of Germany

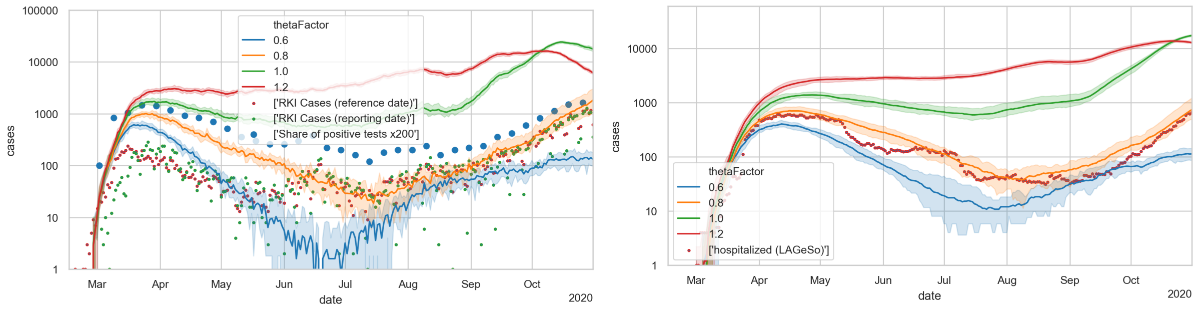

Figure 3: Case numbers (LEFT) and hospital numbers (RIGHT) in simulations that include summer disease import.

available, we ran simulations for the Berlin region (about 5 million inhabitants), the Munich region (about 3 million inhabitants), and an area around Heinsberg (an early outbreak location) including Köln and Düsseldorf (about 7 million inhabitants). In these simulations, we found that interventions that control the virus spread in Berlin did this better in Munich (lower R-value), and even better in Heinsberg. Because of the urgency to have policy-relevant insights, it was thus decided to concentrate on the Berlin simulations, which are for that reason the basis of the present paper.

A simulation of the full Berlin region takes approximately 60 minutes per simulated month. Since we had originally started with a 25% sample of the full population and had good experiences with that (cf. Details of the mobility model), it was decided to continue with that. It reduces the computing time to 15 minutes per simulated month. The runs on our website typically explore about thousand different scenarios; they are run in parallel on a high performance computing system.

It is also feasible to run simulations of all of Germany, but with the present model takes about 16 hours per simulated month. This was considered too slow for the investigation of policy options. It is possible to much accelerate the code by parallelization; this will be considered in future work.

References

[1] Destatis. Privathaushalte nach Haushaltsgröße im Zeitvergleich; 2020. https://www.destatis.de/DE/Themen/Gesellschaft-Umwelt/Bevoelkerung/Haushalte-Familien/Tabellen/1rbev05.html.

[2] Streeck H, Schulte B, Kuemmerer B, Richter E, Hoeller T, Fuhrmann C, et al. Infection fatality rate of SARS-CoV-2 infection in a German community with a super-spreading event. medRxiv. 2020;doi:10.1101/2020.05.04.20090076.

[3] Robert Koch-Institut. COVID-19-Dashboard; 2020. www.corona.rki.de.

[4] an der Heiden M, Hamouda O. Schätzung der aktuellen Entwicklung der SARS-CoV-2-Epidemie in Deutschland – Nowcasting; 2020. Available from: https://dx.doi.org/10.25646/6692.

[5] Berlin L. Time series of hospitalized patients in Berlin;. Available from: https://data.lageso.de/lageso/corona/corona.html#station%C3%A4re-behandlung.

[6] Wikipedia contributors. COVID-19-Pandemie in Berlin; accessed 2020-11-05. https://de.wikipedia.org/w/index.php?title=COVID-19-Pandemie_in_Berlin&oldid=205195302.

[7] BVG. Willkommen bei den Berliner Verkehrsbetrieben | BVG; 2020. https://www.bvg.de/de.

[8] Robert Koch Institut. Kontaktpersonen-Nachverfolgung bei respiratorischen Erkrankungen durch das Coronavirus SARS-CoV-2; 2020. https://web.archive.org/web/

20201018130652/https://www.rki.de/DE/Content/InfAZ/N/Neuartiges_Coronavirus/Kontaktperson/Management.html.

[9] tagesschau. Bund und Länder einigen sich auf weitreichende Lockerungen; 2020. https://www.tagesschau.de/inland/lockerungen-bund-laender-101.html.

[10] Berliner Zeitung. Corona: Berlin überlastet bei Kontakt-Nachverfolgung; 2020. https://www.berliner-zeitung.de/news/corona-berlin-ueberlastet-bei-kontakt-nachverfolgung-li.110998.

[11] Ziemke D, Kaddoura I, Nagel K. The MATSim Open Berlin Scenario: A multimodal agent-based transport simulation scenario based on synthetic demand modeling and open data. Procedia Computer Science. 2019;151:870–877. doi:10.1016/j.procs.2019.04.120.
