# Supplementary material for: Predicting the effects of COVID-19 related interventions in urban settings by combining activity-based modelling, agent-based simulation, and mobile phone data
Source: PLoS One. 2021 Oct 28;16(10):e0259037. doi: 10.1371/journal.pone.0259037 (PMC8553173; doi:10.1371/journal.pone.0259037)
Supplement: S1 Fig — (PDF) [file pone.0259037.s002.pdf]

S1 Figure. Reduced activity participation by activity type

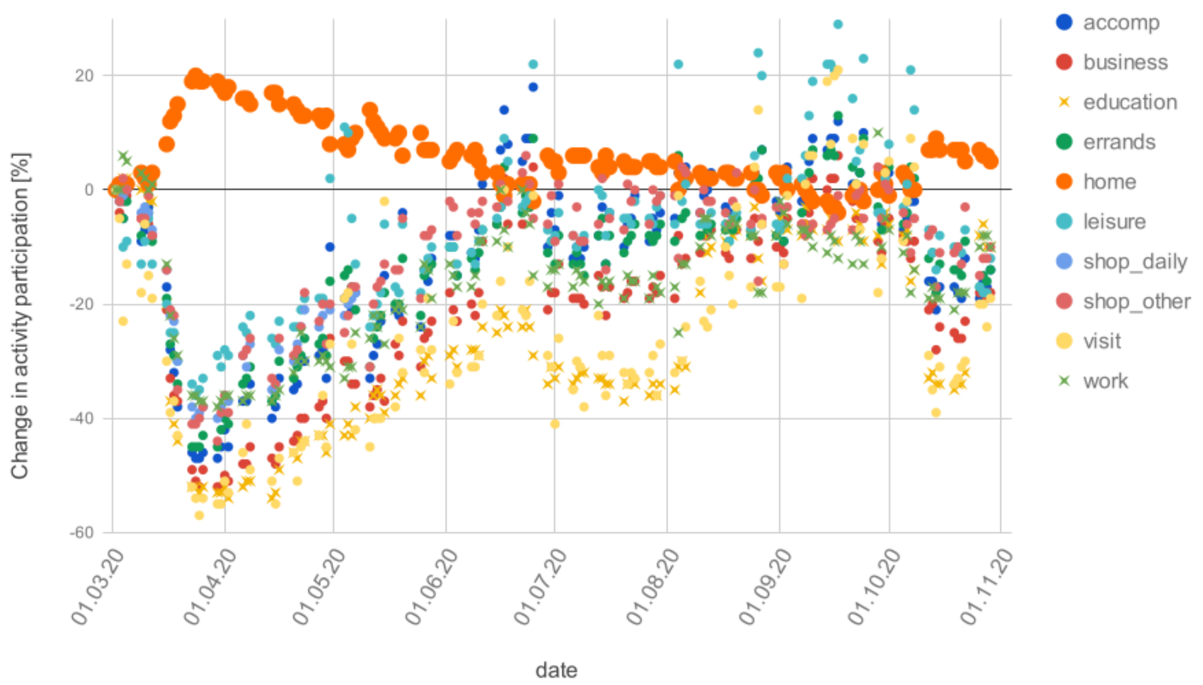

Figure 1: Reduced activity participation by activity type over the course of the epidemics in Berlin as returned by the senozon method. As discussed in the text, the activity types are reliable during periods with normal activity participation, but they are biased during periods with reduced activity participation, as can be seen from the "education" activities, which were closed from 2020-04 until 2020-07 exept for emergency services but where the figure shows time-varying activity participation.
