# Supplementary material for: Predicting the effects of COVID-19 related interventions in urban settings by combining activity-based modelling, agent-based simulation, and mobile phone data
Source: PLoS One. 2021 Oct 28;16(10):e0259037. doi: 10.1371/journal.pone.0259037 (PMC8553173; doi:10.1371/journal.pone.0259037)
Supplement: S1 Text — (PDF) [file pone.0259037.s003.pdf]

S1 Text. Senozon method

Since we are interested in trajectories for large population samples, we start from the network-based approach. Because of privacy restrictions in Germany, the trajectories, even when cut into 8-hour segments, cannot be used directly. In order to be compliant with existing privacy regulations in Germany, they are therefore processed before they can be used for scientific work. The steps are [1], cf. Fig 1 below:

1. First, a synthetic population with attributes home location, age, gender, and employment status is generated based on available census data.
2. Separately, the signalization records, i.e. of celltower handovers, are converted into plausible movement trajectories. This step attempts to remove celltower handovers that occur because of operator load rebalancing rather than user movements, and to remove celltower handovers that occur during travel (since the method is only interested in activities).
3. The resulting trips between activities are exported as plain hourly origin-destination matrices.
4. At the same time, the movement trajectories from step 2 are annotated with demographic categories from the Customer Resource Management (CRM) system of the cellphone company, and with plausible activity types, based on land-use properties of the celltower areas and time-of-day while the locations of the detected activities are removed.
5. For each synthetic person from step 1, at least 30 patterns from step 4 are selected, based on closeness between attributes of the synthetic person and attributes of the pattern owner; one of the patterns is selected randomly. The result, i.e. the synthetic person with his/her activity pattern including their times is exported as well.
6. The resulting activity pattern is enriched with location information for all non-home activities based on the travel times between activities and the origin-destination matrix from step 3. Any trips that use public transport are, based on public transit schedules, assigned to a specific schedule-based connection.

Our second data set, which contains the reductions of activity participation data for each day since 2020-03-02 (cf. Fig 7 in main article and S1 Fig), is derived in the same way, but the computation is stopped after step 2, and only the average times spent at each activity type are aggregated per zip code and then exported.

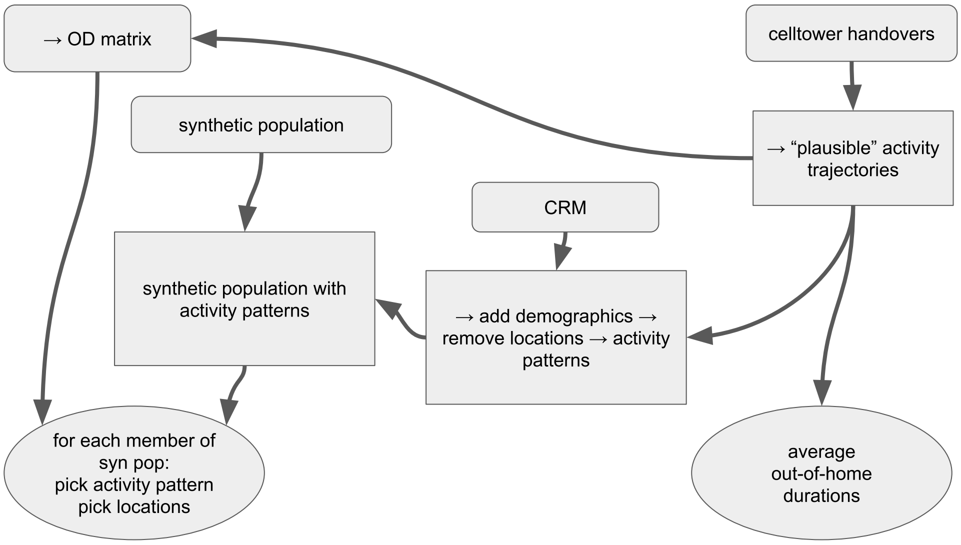

Figure 1: Data flow of the senozon method. Rectangles with rounded corners denote inputs; ellipses denote the two outputs: Synthetic trajectories (bottom left) and out-of-home durations (bottom right). OD matrix = origin destination matrix; CRM = Customer Relationship Management.

References

[1] Neumann A, Balmer M. Mobility Pattern Recognition (MPR) und Anonymisierung von Mobilfunkdaten. Senozon Deutschland GmbH and Senozon AG; 2020. Available from: https://senozon.com/wp-content/uploads/Whitepaper_MPR_Senozon_DE.pdf.
