# Supplementary material for: Predicting the effects of COVID-19 related interventions in urban settings by combining activity-based modelling, agent-based simulation, and mobile phone data
Source: PLoS One. 2021 Oct 28;16(10):e0259037. doi: 10.1371/journal.pone.0259037 (PMC8553173; doi:10.1371/journal.pone.0259037)
Supplement: S2 Text — (PDF) [file pone.0259037.s004.pdf]

S2 Text. Model history

The first version of our model used uniform contact intensities everywhere except in public transport (with $ci = 10$), and in consequence predicted infections mostly where people spend time with other people. Using this model, we showed that even with a complete lockdown, it takes more than a week for infection rates to drop significantly because infections continue to occur at home [1].

A major step then was to integrate actual reductions of mobility behavior based on mobile data into the model. A key finding from using this data was that the population had already changed its behavior significantly before the restrictions were introduced. That model predicted that a 50% reduction in leisure activities would reduce the reproduction number R by 15% while a 50% reduction in work activities would reduce R by 19%. A reduction of all out-of-home activities by 50% resulted in a reduction of R by 62% [2].

In the next step, we integrated seasonality into the model. Thus, more contacts take place indoors in winter than in summer, so that the reproduction number is higher in winter than in summer. With this step, our model was able to correctly represent the second wave in Berlin. We integrated contact intensities per activity type [3], which are based on room size and air exchange rate. The model is therefore based on the assumption that infections mainly occur via aerosols in the air [4]. We also included lower susceptibility and infectiousness of children as was by then reported in the literature [5, 6]. We used this model to show what contributions different activity types have for the overall dynamics [7].

References

[1] Müller SA, Balmer M, Neumann A, Nagel K. Mobility traces and spreading of COVID-19. medarxiv. 2020;doi:10.1101/2020.03.27.20045302.

[2] Müller SA, Balmer M, Charlton B, Ewert R, Neumann A, Rakow C, et al. Using mobile phone data for epidemiological simulations of lockdowns: government interventions, behavioral changes, and resulting changes of reinfections. medRxiv. 2020;doi:10.1101/2020.07.22.20160093.

[3] Kriegel M, Buchholz U, Gastmeier P, Bischoff P, Abdelgawad I, Hartmann A. Predicted Infection Risk for Aerosol Transmission of SARS-CoV-2. medRxiv. 2020;doi:10.1101/2020.10.08.20209106.

[4] Marr et al . FAQs on Protecting Yourself from aerosol transmission; 2020. `https://tinyurl.com/FAQ-aerosols`.

[5] Dattner I, Goldberg Y, Katriel G, Yaari R, Gal N, Miron Y, et al. The role of children in the spread of COVID-19: Using household data from Bnei Brak, Israel, to estimate the relative susceptibility and infectivity of children. PLOS Computational Biology. 2021;17(2):1–19. doi:10.1371/journal.pcbi.1008559.

[6] Davies NG, Klepac P, Liu Y, Prem K, Jit M, CMMID COVID-19 working group, et al. Age-dependent effects in the transmission and control of COVID-19 epidemics. Nat Med. 2020;26(8):1205–1211. doi:10.1038/s41591-020-0962-9.

[7] Müller SA, Balmer M, Charlton W, Ewert R, Neumann A, Rakow C, et al. A realistic agent-based simulation model for COVID-19 based on a traffic simulation and mobile phone data; 2020. Available from: `https://arxiv.org/abs/2011.11453`.
