# Supplementary material for: Predicting the effects of COVID-19 related interventions in urban settings by combining activity-based modelling, agent-based simulation, and mobile phone data
Source: PLoS One. 2021 Oct 28;16(10):e0259037. doi: 10.1371/journal.pone.0259037 (PMC8553173; doi:10.1371/journal.pone.0259037)
Supplement: S3 Text — (PDF) [file pone.0259037.s005.pdf]

S3 Text. Error metric for calibration

As stated, we have decided to use RMSLE as metric to compare hospital numbers from our simulation against real data. RMSLE has the advantage that it is less sensitive to the scale of the data than RMSE. That is, relative errors in valleys have as much weight as relative errors on the ridges. This also corresponds to the visual impression of the logarithmic plots often used in epidemics and used throughout the paper. In a review article by Hazelbag et al [?], the majority of papers uses an absolute error measure such as "squared distance". However, there is also a fair number of papers that uses "relative distance". Also, calibration by "visual inspection", an often-used informal approach, will result in a logarithmic error message when using a logarithmic plot. For comparison, we performed the calibration with RMSE as error metric instead, as shown in Fig. 1 and Table 1 below. Because RMSE is sensitive to the scale of the data, the error is weighted relativly higher during periods of high case numbers, such as during the peak of the first wave. Because of that the fit is better during these times, but less close to actual numbers during the summer. This is particularly visible in Fig. 1 right, which means that if we know (or guess) the actual activity levels, then using RMSE results in a fit that is considerable worse than using RMSLE.

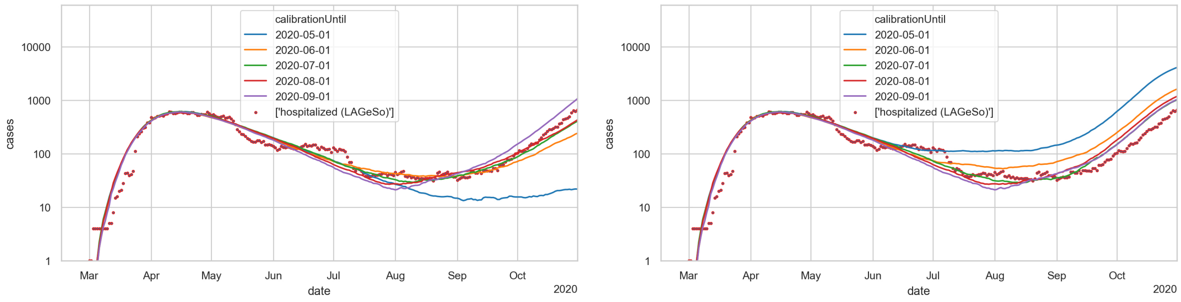

Figure 1: Hospitalized persons for different calibration runs compared to real data. Same plot as Fig 17 of the main text, except that RMSE insted of RMSLE is used as the error metric. LEFT: Activity levels are frozen at the level of the last day of the period used for calibration – the predictive capability is similar than when using RMSLE (Fig 17 of the main text left) RIGHT: Real activity levels are used – the model is now visually considerably worse than when using RMSLE (Fig 17 of the main text right).

Table 1: Θ and activity participation for the different out of sample predictions shown in Fig 17 of the main text. This is the same setup as in Table 2 of the main text except that RMSE (= Root Mean Squared Error) metric is used for the calibration interval (training error) as well as for prediction period between 09-01 and 10-31 (prediction error). The values here are not comparable to the values in Table 2 of the main text because different error metrics are used.

run	Θ	activity participation (if activity level frozen)	training error	prediction error (frozen activity levels)	prediction error (real activity levels)
2020-05-01	1.267e-05	71%	38.62	232.33	1443.54
2020-06-01	1.288e-05	88%	51.74	140.26	406.03
2020-07-01	1.292e-05	90%	49.95	83.51	161.97
2020-08-01	1.267e-05	90%	46.80	75.59	228.81
2020-09-01	1.295e-05	96%	42.38	138.17	167.64
