# Supplementary material for: Predicting the effects of COVID-19 related interventions in urban settings by combining activity-based modelling, agent-based simulation, and mobile phone data
Source: PLoS One. 2021 Oct 28;16(10):e0259037. doi: 10.1371/journal.pone.0259037 (PMC8553173; doi:10.1371/journal.pone.0259037)
Supplement: S4 Text — (PDF) [file pone.0259037.s006.pdf]

S4 Text. Robustness runs

In this section, we proceed similarly to the previous section, but (a) remove the mask model for public transport and shopping activities, and (b) move the spring threshold temperature for moving outdoors from 17.5 to 20°C. Since these changes also affect the training data, Θ is recalibrated under these new conditions.

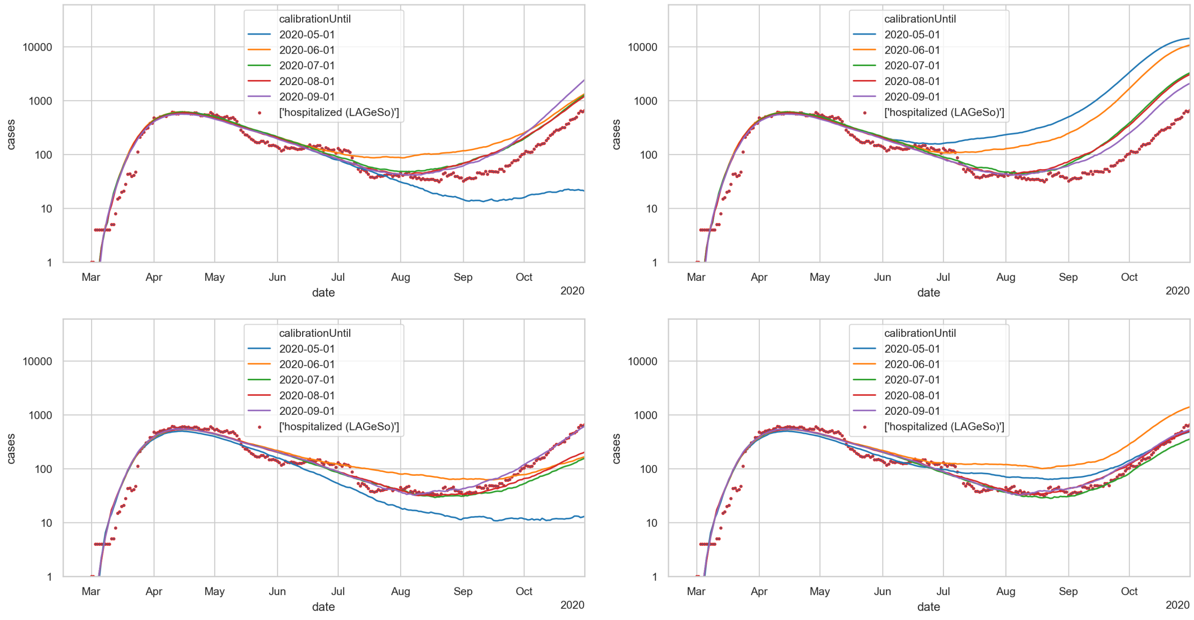

Figure 1: Hospitalized persons for different calibration runs compared to real data. Θ is calibrated such that hospital numbers in the simulation match the real data (red dots) until different points in time as indicated by the legend. After this date, an out of sample prediction is carried out. Until the calibration date, real weather and disease import data is used. After the calibration date, average weather data from the past ten years is used and the disease import is set to 4 imported cases per day (= 1 randomly infected agent per day for our 25% sample). TOP row: Removed mask model. BOTTOM row: Spring threshold temperature changed from 17.5 to 20°C. LEFT column: Activity levels are frozen at the level of the last day of the period used for calibration. RIGHT column: Real activity levels are used. – Results are averaged over 30 Monte Carlo seeds.

Table 1: Calibration parameter Θ and activity participation for the different out of sample predictions shown in Fig 1. RMSLE (= Root Mean Square Logarithmic Error) for the calibration interval (training error) as well as for prediction period between 09-01 and 10-31 (prediction error). For the errors, first the hospital case numbers are averaged over all 30 Monte Carlo runs, and then the errors are computed.

run	Θ	activity participation (if activity level frozen)	training error	prediction error (frozen activity levels)	prediction error (real activity levels)
2020-05-01	1.27e-05	71%	0.322	4.027	10.905
2020-06-01	1.27e-05	88%	0.266	0.962	7.312
2020-07-01	1.27e-05	90%	0.208	0.500	1.815
2020-08-01	1.26e-05	90%	0.171	0.500	1.660
2020-09-01	1.25e-05	96%	0.158	0.867	0.918
2020-05-01	1.11e-05	71%	0.355	5.624	0.169
2020-06-01	1.18e-05	88%	0.269	0.448	1.104
2020-07-01	1.17e-05	90%	0.212	0.609	0.072
2020-08-01	1.18e-05	90%	0.188	0.369	0.048
2020-09-01	1.17e-05	96%	0.154	0.035	0.051

When not using masks (Fig 1 top), it becomes more difficult to calibrate the model. Since masks in public transport/shopping were not introduced before the end of April, the 2020-05-01 calibration does not include the effect of the missing masks, and the different value of Θ can

rather be seen as an indicator of the confidence interval of the calibration. For all later calibration end dates, the missing masks mean that Θ is smaller than before to bring infection numbers down. As one can then see in the out-of-sample runs, especially with the real activity levels (Fig 1 top right), Θ is not brought down enough, since also the period before the introduction of masks is part of the calibration, and in consequence the second waves starts too early and goes up too high.

Calibrating the model also becomes more difficult when changing the spring threshold temperature (Fig 1 bottom). With a higher spring threshold temperature, the effect of moving activities outdoors is reduced, and the calibration needs to compensate by using a smaller Θ.
