# Supplementary material for: Predicting the effects of COVID-19 related interventions in urban settings by combining activity-based modelling, agent-based simulation, and mobile phone data
Source: PLoS One. 2021 Oct 28;16(10):e0259037. doi: 10.1371/journal.pone.0259037 (PMC8553173; doi:10.1371/journal.pone.0259037)
Supplement: S5 Text — (PDF) [file pone.0259037.s007.pdf]

S5 Text. Comparison to other models

Comparison to compartmental models Arguably, compartmental models are the mainstay of epidemiological modelling. Our approach, in contrast, follows individual synthetic persons. These individual persons can be enriched by person-centric attributes such as age or individual risk factors. Disease progression is individual, taking into account these demographic and other person-centric attributes. Similar to compartmental models, the base reproduction number and the starting date need to be calibrated from case numbers (for the present study, the latter was replaced by data-driven disease import). However, both the spatial and the social interactions in our model come directly from data. Also, behavioral reductions in activity participation come directly from data. Mechanical aspects such as the wearing of masks by certain persons and/or at certain activity types can be integrated very simply into the model, by reducing virus shedding, virus intake, or both. Travel in public transport is already integrated. Organizational suppression approaches, such as contact tracing, can be simulated mechanically, thus extracting information about the allowed delays between symptom onset and reaching contacts, the failure rate, etc.

We were able to bring this model up quickly: Coding of the infection code was started at the end of Feb/2020; our first preprint is from 20/Mar/2020 [1]; our first report to the government is from 8/Apr/2020 [2]; we have reported to the government regularly since then.[1] Evidently, we were drawing from our experience and expertise with person-centric travel models. Still, it means that given the right experience and data availability, the method is not overly heavyweight, and once implemented has many advantages over compartmental models.

The basic behavior of the model is like that of any S(E)IR model, i.e. exponential growth until a sufficient share of the population is immune, followed by exponential decline (cf. blue line in Fig **??**). Also, the beginning and the speed of the growth are calibrated in similar ways. In typical S(E)IR models, however, interventions such as reductions in out-of-home activity participation, masks, or contact tracing, need to be parametrized into parameter changes of the S(E)IR model, most notably the infection rate [3, 4, 5, 6]; in our model, such interventions are included directly in the corresponding processes.

A model that is at the border between compartmental and agent-based is by Chang et al. [7]. Important differences to our model include:

- Chang et al. take their movement model directly from mobile phone data. We, in contrast, reuse a pre-existing, activity-based model from transportation planning. This may be an advantage in regions where such a model already exists, and in particular so if the differentiated data that Chang et al. have is not available (as seems to be the case in Germany).
- We can attach individual attributes to each agent. In the present paper, this is used to model age dependence, a future study will contain the virus mutations, but it could also be used to include, say, pre-existing conditions. Compartmental models can only achieve this by introducing partial densities in each department. They will need as many partial densities as there are attribute combinations, i.e. $N_{ageGroups} \cdot N_{mutations}$ for the above situation. Since this is also multiplied with the number of locations, such models eventually need (much) more memory than when the information is attached directly to the individual agents.
- Both models are similar in that the conditional infection probability given contact is indirectly proportional to the floor area, for us stated in Eq. (**??**), and for Chang et al. stated in Eq. (8) in the appendix. We take floor area from maximum occupancy (obtained from the person trajectories) plus the typical surface area per person for each activity type. Chang et al, in contrast, take the floor area directly from their POI data. However, they do not explicitly consider workplaces or schools, and rather replace this by a parameterized "localized infection"; here, our model is more specific and thus more conducive to the consideration of explicit measures such as the introduction of masks, reduced activity

[1] Cf. `https://depositonce.tu-berlin.de/simple-search?query=modus-covid`

participation, etc.
Our model also takes typical air exchange rates into account.

- Our model, since it comes from transport planning, includes encounters in public transport vehicles.
- Another difference, which, however, has nothing to do with the methodology, is that we calibrate and validate against hospital cases. This provides, at least in Berlin, a more stable basis than case numbers, since in Germany the sampling strategy for PCR testing has changed several times, thus making the case numbers problematic as a time series.

Comparison to other agent-based models driven by mobile phone data As stated earlier, an approach similar to ours is by Aleta et al. [8]. There are the following differences:

- Aleta at al, similar to Chang et al, take their movement model directly from the mobile phone data. We, in contrast, use a pre-existing model from transportation planning. This is useful in particular in places where such a model already exists.
- Importantly, we also use the reduction-of-mobility data as input to our model, which they do not.
- Aleta at al. use a model of 2% of the real population (85,000 synthetic persons) while ours consists 25% (1.25 mio). With models sampled at fraction α, one needs to make a decision if either one synthetic person stands for $1/\alpha$ real persons, or if one models a fraction of the real population. We found the second path more intuitive. However, one needs to make sure that household sizes, group sizes at offices, etc., remain realistic. For household sizes, this can be achieved by synthetically constructing them, as both Aleta et al. and we do in some way. For all other locations, one needs to aggregate $1/\alpha$ locations of the same type into one location in order to have realistic contact probabilities. Aleta at al. state that because of the sampled population, "colocation events between individuals are still quite sparse", which points exactly to that issue.
- One issue with small population samples is that this makes the introduction of different virus strains difficult: One can essentially only introduce them in packets of $1/\alpha$, missing the relatively long early phase where their numbers are still low. We will report on this in a future paper.
- In contrast to Aleta et al, we use an infection model constructed from first principles. This would presumably be easy to change in their model.
- Where we use lognormal distributions, Aleta et al. use exponential distributions (i.e. rates) to transition from one disease state to the next; we believe that the literature prefers lognormal distributions. Using exponential distributions will lead to wider distributions of disease durations, both with shorter and longer durations from infection to recovery. This also would presumably be easy to change in their model.

References

[1] Müller SA, Balmer M, Neumann A, Nagel K. Mobility traces and spreading of COVID-19. medarxiv. 2020;doi:10.1101/2020.03.27.20045302.

[2] Müller SA, Charlton W, Ewert R, Rakow C, Schlenther T, Nagel K. MODUS-COVID Vorhersage vom 8.4.2020. depositonce. 2020;doi:10.14279/DEPOSITONCE-10016.

[3] Dehning J, Zierenberg J, Spitzner FP, Wibral M, Neto JP, Wilczek M, et al. Inferring change points in the spread of COVID-19 reveals the effectiveness of interventions. Science. 2020;doi:10.1126/science.abb9789.

[4] Eichner M, Schwehm M. CovidSIM; accessed 2020-08-27. `http://covidsim.eu/`.

[5] Neher R, many others. COVID-19 Scenarios; accessed 2020-08-27. `https://covid19-scenarios.org/`.

[6] Althaus C. Real-time modeling and projections of the COVID-19 epidemic in Switzerland; 2020. https://ispmbern.github.io/covid-19/swiss-epidemic-model/.

[7] Chang S, Pierson E, Koh PW, Gerardin J, Redbird B, Grusky D, et al. Mobility network models of COVID-19 explain inequities and inform reopening. Nature. 2020;doi:10.1038/s41586-020-2923-3.

[8] Aleta A, Martín-Corral D, Pastore Y Piontti A, Ajelli M, Litvinova M, Chinazzi M, et al. Modelling the impact of testing, contact tracing and household quarantine on second waves of COVID-19. Nat Hum Behav. 2020;doi:10.1038/s41562-020-0931-9.
