# Supplementary material for: Predicting the effects of COVID-19 related interventions in urban settings by combining activity-based modelling, agent-based simulation, and mobile phone data
Source: PLoS One. 2021 Oct 28;16(10):e0259037. doi: 10.1371/journal.pone.0259037 (PMC8553173; doi:10.1371/journal.pone.0259037)
Supplement: S6 Text — (PDF) [file pone.0259037.s008.pdf]

S6 Text. Making the model more realistic

Adaptation of daily schedules to restrictions In the current model, if an activity is selected for removal because of reduction of activity participation, then that activity plus the preceeding and succeeding trip are simply deleted from the schedule. The schedule is not repaired accordingly: neither is the home activity nor are other activities prolonged, and also the trip chain is not mended. This could be improved by using a behavioral model that explicitly reacts to these changes. The established framework of activity-based modelling in transportation (e.g. [1, 2, 3, 4, 5]) could be used for that; for a first step in that direction in the context of COVID-19 see [6]. For our present model, we believe that this does not have a high priority since our activity participation is data driven: If people compensate by prolonging other activities, this will show up in the data and in the consequence in our model. The issue would become more urgent if the prediction of behavioral responses became part of the model.

Income It has been stated that income plays an important role for the risk of becoming infected. Possible pathways via which this can occur include jobs that puts people more at risk, lack of capability to follow detailed instructions possibly because of language issues, or less space per person at home. In particular that last item could be added into the simulation, e.g. by varying the floor area per person at home in Table 6 of the main text according to the affluence of a given spatial area – this would have direct consequences in our model for the reproduction number at home.[1] Also, since our data is resolved to zipcode level, it would be possible to replace the input of Fig 7 of the main text by spatially resolved activity participation data. These aspects need to be investigated in future work.

Contact structures It is well established that different contact structures lead to different infection dynamics [7, 8]. For example, the epidemic threshold, i.e. the minimal share of persons that need to be susceptible, may be different. For our model, such elements in principle come from the input data: Besides coming with their complete contact graph, our synthetic persons have an age, an employment status, and gender. Thus, in principle, we have the interaction structure at the level of facilities or public transport vehicles, including people's attributes, from data. However, as stated, for privacy reasons the facilities are too large, and in consequence multiple households or multiple offices are bundled into a single facility. For households we compensate, as described, by manually splitting them up; as of now we do not, however, control for age structure in the splitting process. This needs to be improved. For all other activities we compensate, as also described, by allowing interaction with only $1/N^{spacesPerFacility}$th of all other persons at the same facility on each given day, but allowing mixing by using separate random draws for every simulated day, selecting $N^{spacesPerFacility}$ such that the overall number of contacts (for contact tracing) ends up in a plausible range. This should be improved as well. However, more of an issue for COVID-19 may be that the original input data does not contain separate facilities for the elderly; for mobility modelling, this has so far not been of interest. This implies that the following issues need to be addressed in the future:

- Clarify which epidemiology-relevant aspects of the input data are too far away from reality.
- For those aspects that need to be improved, clarify if this could be done at the level of the original input data generation, or if it should be compensated for at the level of the modelling.

Concerning the possibly different epidemic threshold, we would argue that for the present situation this is less of a problem: If only a fraction of the persons in the simulation was susceptible, or they would be connected via a different contact structure, the calibration process would compensate by selecting a different Θ in Eq. (1). Evidently, if we get closer to herd immunity, possibly by vaccination, these aspects become more important.

[1] In contrast, floor area per person at schools is standardized in Germany.

References

[1] Bowman JL, Bradley M, Shiftan Y, Lawton TK, Ben-Akiva M. Demonstration of an activity-based model for Portland. In: World Transport Research: Selected Proceedings of the 8th World Conference on Transport Research 1998. vol. 3. Elsevier, Oxford; 1998. p. 171–184.

[2] Arentze TA, Timmermans HJP. A learning-based transportation oriented simulation system. Transportation Research Part B. 2004;38:613–633. doi:10.1016/j.trb.2002.10.001.

[3] Bellemans T, Kochan B, Janssens D, Wets G, Arentze T, Timmermans H. Implementation framework and development trajectory of FEATHERS activity-based simulation platform. Transportation Research Record. 2010;2175(1):111–119. doi:10.3141/2175-13.

[4] Bhat C, Goulias K, Pendyala R, Paleti R, Sidharthan R, Schmitt L, et al. A household-level activity pattern generation model with an application for Southern California. Transportation. 2013;40(5):1063–1086. doi:10.1007/s11116-013-9452-y.

[5] Vovsha P. Microsimulation Travel Models in Practice in the US and Prospects for Agent-Based Approach. In: Bajo J, editor. Highlights of Practical Applications of Cyber-Physical Multi-Agent Systems: International Workshops of PAAMS 2017. Cham: Springer International Publishing; 2017. p. 52–68. Available from: `http://dx.doi.org/10.1007/978-3-319-60285-1_5`.

[6] Najmi A, Safarighouzhdi F, Miller EJ, MacIntyre R, Rashidi TH. Determination of COVID-19 parameters for an agent-based model: Easing or tightening control strategies. medRxiv. 2020;doi:10.1101/2020.06.20.20135186.

[7] Pastor-Satorras R, Castellano C, Van Mieghem P, Vespignani A. Epidemic processes in complex networks. Rev Mod Phys. 2015;87(3):925–979. doi:10.1103/RevModPhys.87.925.

[8] Aleta A, Martín-Corral D, Pastore Y Piontti A, Ajelli M, Litvinova M, Chinazzi M, et al. Modelling the impact of testing, contact tracing and household quarantine on second waves of COVID-19. Nat Hum Behav. 2020;doi:10.1038/s41562-020-0931-9.
