# Supplementary material for: Predicting the effects of COVID-19 related interventions in urban settings by combining activity-based modelling, agent-based simulation, and mobile phone data
Source: PLoS One. 2021 Oct 28;16(10):e0259037. doi: 10.1371/journal.pone.0259037 (PMC8553173; doi:10.1371/journal.pone.0259037)
Supplement: S1 File — (PDF) [file pone.0259037.s009.pdf]

Von: **Sebastian Müller** mueller@vsp.tu-berlin.de
Betreff: Re: Urgend Figure permission
Datum: 29. Juni 2021 um 22:21
An: Ewert, Ricardo ewert@vsp.tu-berlin.de

SM

Von: Tim Wakeford <tim.wakeford@ubiquitypress.com>
Gesendet: Dienstag, 29. Juni 2021 10:51
An: Ewert, Ricardo <ewert@vsp.tu-berlin.de>
Cc: info@ubiquitypress.com; Brian Hole <brian.hole@ubiquitypress.com>
Betreff: Re: Urgend Figure permission

Dear Ricardo,

Thank you for getting in touch. As the source material has been published under a CC BY licence, no additional permission is required. You are able to re-use the content in this book (unless individually licenced) without a permission form requirement. CC BY simply requires that you make it clear where the source material has come from by clearly citing the original material. In your preprint, I see that you have already cited the source book, so this requirement has been filled.

One thing that I do ask is that you add the book DOI to your reference. Please could you ensure that the below information is included in the reference, so that the citation is clear and readers can find the material easily. This will also ensure that the two resources link up better via the metadata:

Horni, A., Nagel, K. and Axhausen, K. W. (eds) (2016) The Multi-Agent Transport Simulation MATSim. London: Ubiquity Press. DOI: **https://doi.org/10.5334/baw**

Kind regards,

Tim

Tim Wakeford
Head of Editorial
Ubiquity Press

S: tim.wakeford
W: https://www.ubiquitypress.com

The information contained in this email is intended for the addressee(s) only and may be confidential, legally privileged and/or protected by law, including law of copyright. Unauthorised use, copying, forwarding or disclosure may be unlawful. If you are not the intended recipient, please delete this e-mail and notify the sender immediately, or notify Ubiquity Press via our Privacy Page.

On Mon, 28 Jun 2021 at 14:48, Ewert, Ricardo <ewert@vsp.tu-berlin.de> wrote:

> Hello,
>
> we (research group of Prof. Kai Nagel) request permission for the open-access journal PLOS ONE to publish a Figure of the paper “Predicting the effects of COVID-19 related interventions in urban settings by combining activity-based modelling, agent-based simulation, and mobile phone data” under the Creative Commons Attribution License (CCAL) CC BY 4.0
> (http://creativecommons.org/licenses/by/4.0/)

(http://creativecommons.org/licenses/by/4.0/).
Please be aware that this license allows unrestricted use and distribution, even commercially, by third parties. Please reply and provide explicit written permission to publish the Figure in “Predicting the effects of COVID-19 related interventions in urban settings by combining activity-based modelling, agent-based simulation, and mobile phone data” under a CC BY license and complete the attached form.

Preprint PLOS Paper: https://doi.org/10.1101/2021.02.27.21252583
Original book at Ubiquity Press: MATSim book https://doi.org/10.5334/baw

Figure of needed permission:

- preprint Fig. 1

Original figure:

- MATSim book Fig. 2.2

Best regards,
Ricardo Ewert
--
Research Associate
TU Berlin
Department of Transport Systems Planning and Transport Telematics
www.vsp.tu-berlin.de
vsp.berlin
www.matsim.org
