# Supplementary material for: Predicting the effects of COVID-19 related interventions in urban settings by combining activity-based modelling, agent-based simulation, and mobile phone data
Source: PLoS One. 2021 Oct 28;16(10):e0259037. doi: 10.1371/journal.pone.0259037 (PMC8553173; doi:10.1371/journal.pone.0259037)
Supplement: Attachment — Submitted filename: Response_to_Reviewers.pdf [file pone.0259037.s010.pdf]

Reviewer 1

Reviewer #1: The paper “Predicting the effects of COVID-19 related interventions in urban settings by combining activity-based modeling, agent-based simulation, and mobile phone data” proposes a person-centric model that incorporates mobility patterns derived by cell phone usage and numerous other factors with a person-specific variant of a S(E)IR model to better understand how specific political interventions affect the rate of reinfections. Overall, the model offers an immaculate depth of a possible system that explains the progression of the pandemic in Berlin. Still, there are multiple major and minor questions that I would like to be answered before advising the manuscript for publication.

Major Remarks:
-While I generally admire the depth of the proposed model, I am not completely sure from what empirical evidence we really draw the conclusion. In particular, I am not sure to what extent the model choices or the mobility patterns drive the results. In my opinion, there are still multiple unknowns regarding the transmission of COVID-19, e.g., where do they happen, what effective difference is between an N95, medical mask, or cloth. Still, all this missing knowledge is completed with sometimes more or less empirical values in the presented model. I definitely see the sense of having this type of model, but the caveats should be stated clearly in the paper.

-The article would greatly benefit from a discussion of the sensitivity and stability of the system. If I understand the manuscript correctly, only one parameter is chosen in the Calibration, but how sensitive and stable is the system to different values of this coefficient? And what kind of sensitivity checks of all other parameters were carried out? By this I mean some assessment of how robust the conclusions drawn are.
An adequate discussion and communication of the uncertainties in the model are missing. On multiple occasions, this would benefit the manuscript.

- Fig. 6 TOP: The numbers are really low, hence some uncertainty over multiple iterations of the pandemic would be great. Here, one could, e.g., sample different mobility patterns in the Senozon method? Overall, multiple runs of the simulations should enable some type of uncertainty quantification.

-The calibration of the only non-fixed parameter also has associated uncertainty. Here some additional information on how the calibration is performed is needed. Can one understand it as some type of method of moments, where the coefficient is picked so that the observed time series of cases and hospitalizations is conserved as closely as possible in the simulation model? If so it is unclear one differentiates between primary and secondary priority. Some mathematical formulas in the Annex or Supplementary Material would be helping.

All these comments revolve around the sensitivity/stability/robustness of the system. We have added two sections “Out of sample prediction” and “Robustness runs” into the results part of the paper, and a section “Robustness” into the discussion part of the paper.

The “first/second” priority is clarified as follows: Calibration is performed by visual comparison, with first priority against the time series of the number of hospital patients in Berlin, and with second priority against

the COVID case numbers in Berlin.\footnote{The calibration procedure, as described in \nameref{sec:calibrationAppendix}, is as much about which elements to include at all as about finding the right parameters. ``Second priority'' here means that if calibration against hospital numbers is undecided between two alternatives, then the case numbers are used in addition.} The case numbers are only used with second priority since the screening procedure has been changed multiple times, which means that the resulting time series is not homogeneous and thus not useful for model calibration. In particular, under-reporting in the initial phases was much larger than later. A formal calibration of $\calibrationParam$ can be found in Sec.~\nameref{sec:out-of-sample-prediction}.

The mask specifications were accidentally removed from the main part of the paper; they are now back (under “model details”).

Since this is a fair amount of text, could we please ask the reviewer to find that text in the manuscript? As stated, it can be found in the following sections:

- Model details → Masks
- Results → Out of sample prediction
- Results → Robustness runs
- Discussion → Robustness

- Fig. 4: What do the shaded areas indicate?

We added an explanation to the figure caption.

In the article prediction using the model is named as a possible application. Therefore, some out-of-sample results using historical data are needed.

We added a seperate section “out of sample prediction” under Methods and results

-What are the effects of the multiplicative form in equation (1)? Doesn't this form assume that all components are inherently on the same scale?

We added the following footnote with an explanation: \footnote{If one wanted to use physical units, then one could decompose $sh = \tilde sh \cdot out$ with $\tilde sh$ the, say, number of shedded virus particles per time. out (for a mask on the shedding side), ci, and in would be correction factors, i.e.\ 1 for a standard situation. τ would denote the time duration, so that the result would be the number V of virus particles that were inhaled during that time duration. $\calibrationParam$, or more precisely $1 - \exp(- \calibrationParam \, V)$, would translate that number of virus particles into an infection probability. If that translation was known, one could attempt to calibrate the model from first principles. Practically, we use $\calibrationParam$ as our main calibration parameter.}

Even though the approximation in (2) is only used for illustrative reasons when does the approximation exactly hold?

We added a new figure (Fig. 2) to show when the approximation holds.

Minor Remarks:

-Generally, the presentation of the introduction could be more concise. At the moment, the narrative is based on how prior versions of the model were improved over time with multiple features but it does not become apparent why we even need a person-centric simulation model next to the standard S(E)IR models readily available.

We have shortened the introduction. The ``historical'' part is now in supplemental material. (We believe that it is useful to have that somewhere.)

Also, I would check if all the citations to prior versions of the model are really needed.

We moved the material on model history to supplementary material. We think that it is a useful service to readers to have the differences between the different preprint versions sorted out.

It is also not clearly evident what the section on related work really wants to achieve. A clear motivation of agent-based models would be more than enough but the different types of mobility data should rather be discussed in the Annex.

Several earlier readers of our draft had understood our work as a contribution to "mobility during Corona", and recommended to position it within that literature. We have rearranged and streamlined the section on related work. We believe that the remaining text and references help to position our paper.

-Senozon method: Some illustrative examples with a Figure akin to Fig. 1 would be a good addition to the explanation. Is for each person one mobility pattern sample for each day? If so how is prediction performed, where no observations of mobility patterns are available? Also, the section would be more meaning full if some toy examples of an origin-destination matrix and the raw data could be provided. What percentage of people is included in the raw data? Some caveats on the uncertainty associated with the plausible classifications of activities would be fitting. Finally, to be it is not clear how people assumed to be meeting one another given two specific meeting patterns.

We added a figure for the data flow. The description of the senozon method refers to a senozon white paper of 6 pages with 4 figures (unfortunately in German); here is the link for convenience: https://senozon.com/wp-content/uploads/Whitepaper_MPR_Senozon_DE.pdf . Those four figures contain, we think, what the reviewer would like to see, but it would make the paper considerably longer. We think that this is somewhat out of scope for the present paper since it is a method developed for transportation, but if the reviewer would like to have it for completeness in the supplementary material, we would be happy to provide it.

-While the title of the manuscript suggests that the focus lies on urban areas, in the text, we find repeated comments that complete Germany is modeled as well. Why are only the results for Berlin reported? Does the sample only include urban areas or what data sources are missing for rural areas?

We added a paragraph "Selection of geographical region and computing times" in the Discussion section.

-Does the simulation assume that each home, school etc has the same constant values named in Table 1? Is this reasonable? Even in Berlin, there is some type of income gap, that probably leads to

more space per person in wealthy neighborhoods. How could this affect the findings of the article? A random distribution could be used as an alternative maybe even based on the ZIP code?

We added a paragraph “Income” in the Discussion section.

As a side note, which we consider too far away from the scope for the paper: We find that the infection dynamics is driven by the subpopulation with the highest R-value. In consequence, if at some point the number of infected persons is the same as in reality, and the R-value is the same as in reality, the prediction will be correct, no matter if the infection is spread across the whole population, or concentrated in a subset. (This is also the reason why using the 25% sample works -- essentially, we take a 25% subset of households.) However, evidently knowing these aspects better would make a difference for counter-measures.

-Are the lines in Fig. 4 in- or out-of-sample predictions of the model? And is the blu line the number of people with symptom onset? If so why is it so far of especially in the first few months?

The only way in which we get the simulation consistent with the hospital numbers is that we assume much more under-reporting in the first few months than later. We discuss this under “Under-reporting” in the discussion, but now added some sentences around Fig.4 (now Fig.5) to make that argument, and to refer to that discussion section.

In addition, the temporal path of the R-value (Fig. 7) compared to the officially reported value by the RKI would be great to see and assess the proposed model.

We tried that, see the attached (blue = simulation, red = official value). Problems with the official values include that

- they count imported cases as intrinsic cases (at the very beginning),
- we do not have them separately for Berlin (Berlin regularly reported a 4-day average R value, because of an assumed serial interval of 4 days for covid; however, the 4-day average value suffers from strong periodic behavior because of the weekend/public holidays)
- The R-value is the first derivative of the case numbers, for which we discuss why we find them unreliable and rather calibrate against hospital numbers.

In consequence, we do not think that this is helpful. The two curves are attached for the reviewer; blue = simulation; red = R-value for Germany (i.e. *not* restricted to Berlin).
We can see that that makes our section on the “reproduction number” a bit moot. We would like to keep it anyways, since it leads into the “R-values per activity Type”.

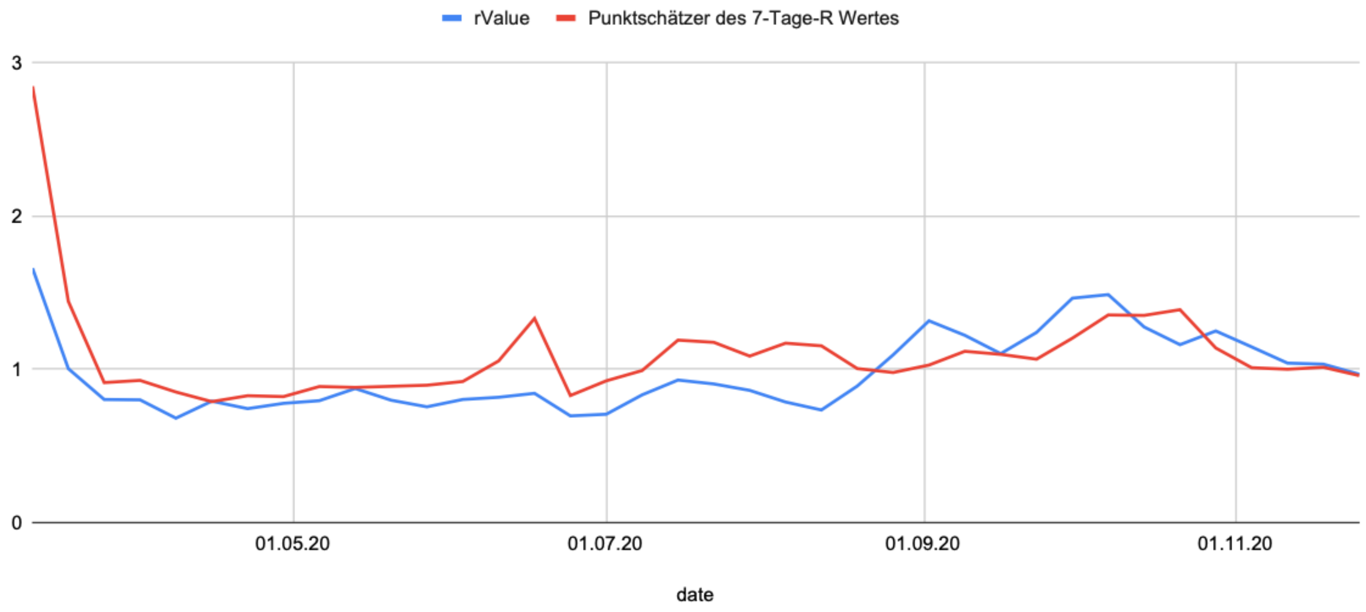

-Did I understand the reasoning behind the additive strucure of interventions correctly in that it is based on the definition as a sum over individual reinfections? If so this should be stated more clearly in the manuscript.

Yes. Thank you for pointing out that this should be explained. We added text as follows:

For example, an activity-specific R-value for school of $R_{school}(t)=0.1$ means that each person that became contagious on day t, in the average, infects 0.1 other persons at school. Evidently, if only 10\% of persons turning contagious on day t have school anywhere in their activity pattern, then each such person would have to infect one other person in the school context in order to reach the population-average value of 0.1.

Adding up these activity-specific reproduction numbers leads to the overall reproduction number. This explains why, in first order, the overall reproduction number can be additively decomposed into the contributions of the different activity types.

-Table 3 is not clear to me. The authors of each study should be directly named together with some classification of the used method. Further, the proposed model should be one column in the table.

Also, the star at 0 is not clear to me, how can 0 be significantly different from 0?

The preprint paper from which this came estimated three different models for "percent reduction of R by different interventions". All three models resulted, for masks, in percent reductions that had error bars that included zero … and that is what we meant: "Paper reports percent reductions of R by masks that are not significantly different from zero". The published version of the paper has the results concerning masks removed, stating "it is difficult to estimate the effect of mask-wearing in public spaces because there was limited public life as a result of other NPIs." We now refer only to the published version of that paper, and in consequence the mask entry, including the confusing footnote, was removed completely.

Reviewer 2

The manuscript “predicting the effects of covid-19 related interventions in urban settings by combining activity-based modeling, agent-based simulation, and mobile phone data” presents a novel approach to simulate the spread of epidemics in urban environments. As opposed to compartmental models, the model presented in the manuscript presents a personalized spatially explicit framework to track the contact between individuals, the probability of infection in each contact, and the progression of the disease in those individuals who were infected. The model manages to predict the trend of the disease spread in the Berlin metropolitan area. As such, I enjoyed reading the manuscript, as it presents a breakthrough in modeling epidemics, by allowing the insertion of explicit and detailed policy measures.
However, the manuscript contains pitfalls and is often unclear. The authors must take this into account in order for the manuscript to make a valid and clear scientific claim regarding the spread of epidemics in urban environments.

Model fit:
The calibration process of the model manages to fit the model with the general trend of the number of cases and hospitalized patients in berlin. However, the manuscript doesn’t suggest any metric that evaluates the model fit. Even if this kind of metric is not the purpose of the authors’ model evaluation, figure 4 presents the data in a logarithmic scale, in order to show the fit of general trend. However, this type of presentation is a bit deceiving without explicitly informing the reader that the purpose of the figure is to compare the trend and not absolute numbers.

It is correct that we have visually compared simulation runs and real world numbers; we now state this more clearly (in Methods and Results → Calibration).

We have now added the following sections:
- Method and Results → Out of sample prediction
- Methods and Results → Robustness runs
- Discussion → Robustness

The formal calibration uses Root Mean Square Logarithmic Error. This indeed corresponds to the visual impression of errors in logarithmic plots, i.e. aiming for constant relative rather than constant absolute error. From a perspective of “saving lives” this is admittedly wrong. From the view of the dynamics, this is correct: A 10% error on infection numbers in some submodel causes a multiplicative error, not an additive error. We know from our own personal experience how difficult it was to switch between multiplicative thinking (for the modelling part) and additive thinking (for the policy advice). We would prefer to leave this aspect out of this already rather heavyweight paper.

Simulating agents activity:
It is clear that the COVID-19 pandemic changed the travel habits of nearly the entire population, in both short-term and long-term habits. It seems that the simulation conducted takes into account only the short-term travel habits, as it does not simulate replanting of agents' plans of mode choice and destination choice, such as changing the activities to telecommuting and teleducation. Choosing a different mode of transport is very likely given lower capacity or frequency in PT systems, and it is possible to rerun the simulation every n days in order to observe what happens to

the modal split. It is true that simulating destination choice is very complex and might not be possible using this framework, however the authors should mention this limitation.

In addition, it is not clear from the paper what happens when a person's activity is removed - how does the rest of its plan change?

We added the below explanation in paragraph "Reduction of activity participation" in section Time-dependent inputs and calibration. Please also see "Adaptation of daily schedules to restrictions" for some discussion on this topic.
"For example, if a person in their original plan goes from home to activity A and then back home, then the activity plus both trips are removed from the schedule. If a person in their original plan goes from home via activity A to activity B and then back home, and activity A is deleted, then the following elements are removed: (a) the trip from home to activity A; (b) activity A; (c) the trip from activity A to activity B. In the current model, the schedule is not repaired: neither is the home activity nor are other activities prolonged, and also the trip chain is not mended."

Section - Infection model:
1. page 8: Equation 1 is not clear enough. M is the sum of which other persons? Those in contact? The explanation is for 2 individuals or for multiple individuals?

Sorry. Yes, m goes over all other persons with which the considered person n has contact with. For the aerosol model that will be used, these are simply all other persons in the room, but other contact models can be imagined.

Eq. (2) indeed assumes a single contagious person in the room, and so we added that as a specification.

2. CI is different for each type of activity, due to the fact that people exhale and inhale more air in different activities (talking, eating, working out etc.). Was this taken into account? Though it affects the wedding rate(sh) more than the actual contact intensity, it is reasonable to materialize it through CI.

We added a sentence as follows where the contact intensities are treated: "Aspects such as loudness of speech or if persons perform a physical activity are not taken into account in the present model although they are known to play important roles [55,56], and they could, to some extent, be attached to the activity types. It is planned to include them in a future version of the model.". The model described in the paper is the production model that we use currently; we would prefer to leave it in that way for the present paper.

Page 9:
The explanation for room capacity was hard to grasp. If I understand correctly, nspaces reflects the fact that there is a smaller chance for two people to meet, however if they do, the chance of infection is higher.

There is now an additional sentence in "Estimation of room sizes" section (in blue):

As stated above, our data resolves down to the level of "facilities". These correspond roughly to buildings. In consequence, such a facility can be anything from a single family home to a large office building or a sports arena. Since these facilities are too large compared to typical rooms, we divide facilities into Nspaces

rooms. Nspaces is set to 20; the argument for this number is given in Handling of large facilities in the appendix.

Most of that material in blue was already there a couple of lines further down but it was hard to see how these belonged together. That material further down is now (evidently) removed.

The intuition that dividing the facilities has no effect in first order is exactly right. We added a paragraph at the end of Model Details → Infection Model → Air exchange rate.

Page 10:
1. Table 1 is unclear. What term does each column come to represent? The air exchange low and high calculation is not detailed in the main body of the text, and is unclear.

This was written in terms of the “air volume flow per person”, since that is used by our colleague Kriegel, and has some theoretical advantages. But it made the text more complicated so we now reverted to describe it in terms of the “air exchange rate”. These we estimate (from literature) for “old” and “new” building and vehicle types, compute resulting contact intensities separately (as stated in the table caption), and then take a weighted average. Hopefully that clarifies it.

2. The calculation of CI needs to be refined in an orderly fashion. I tried to manually calculate the values in table 1 and failed.

We had tried to align our work with Kriegel’s work, which formulated the “risk” of a space as a function of “air volume flow per person”. Given that that made the computation more complicated than necessary, we moved back to the “air exchange rate” ae, which is the rate with which the air in the whole room is exchanged. The result is that ci now simply is = 1/(rs*ae), where rs is the room size. rs in turn is obtained in the model from the maximum number of persons that are ever in the room, Nmax, multiplied with a typical floor space per person, fs. This can thus be expanded as

$$ci = 1/(rs*ae) = 1/(Nmax * fs * ae) = 1/Nmax * 1/(fs*ae) =: 1/Nmax * ci'$$

The “reduced” contact intensity ci’ is thus a property of the room type, while the room size is contained in Nmax. This should make the table easier to understand. Note that all ci’ are given relative to ci’_home.

3. What is the transition probability from being infectious to showing symptoms? It does not appear in the text.

Thanks a lot for pointing that out. We added it into Fig.3 RIGHT. This is a bit overkill since (for the current model) it is the same for all age groups, but in that way it is easy to detect

Sub section - Indoors/outdoors and second wave:
Page 13:
Did leisure activities in Berlin in fact take place outdoors, or that they were also restricted?

We added a paragraph as follows:
There were some restrictions concerning leisure activities in place in fall. They mostly concerned large events. We know from our mobility data that all activities were at their normal level in September 2020; in consequence, if anything, they were divided into smaller groups.

“Too early” “not steep enough” please refer to the specific location in the graph, by annotating the graph or by explicitly mentioning the coordinates on the graph.

We added a more expressive caption to figure 9.

What fraction of the activities do leisure activities take up?

See Discussion → Intuition for these results Table 5: On average, a person spends approximately 1.67 hours / per day at leisure activities. Only at work (1.83 hours / day) and at home (17.92 hours/day) more time is spent.

Sub section - Infections per activity type:
Page 13:
The share of each activity frequency in both count and total duration across all agents is important in order to fully understand figure 6.

There is now a table in Discussion → Intuition for these results, that contains that information. And the text, that was mostly already there before, now refers to that table.

Sub section - Reductions of R per intervention:
Page 16:
Table 2 - is R calculated over the entire period? A certain date? If so please specify when.

We added the following into the figure caption:

For these calculations we ran the unrestricted model without any interventions and then introduced the interventions described in the left column on 2020-04-01. The reductions to the R values were calculated one week after that, comparing the respective weekly averages.

When reporting reference [3], you used percent reduction, however here you use additive reduction while explaining why it is better - you should point out the weakness in percent reduction when first mentioning it(page 2).

We re-wrote the corresponding sentences in the introduction as:

We show how most activity types generate over time fairly constant contributions to the reproduction number R, independent from the actual \emph{level} of R. In consequence it is structurally more stable to report reductions of R caused by interventions as an additive term, rather than a term that is relative to the overall level of R as is usually done (e.g.~\cite{Brauner2020-vf}).

Sub section - Intuition for these results:
Page 17:
The intuition for decreasing marginal effect of interventions should be presented in a formal way. A graphical explanation of intuition for the results would improve the understanding of that part.

We revised the text in Methods and results → Decreasing marginal effect of interventions and added the following figure:

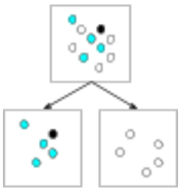

General remarks regarding the organization of the paper:
I don’t fully understand the motivation of the authors to move the calibration section to the appendix. It seems that the calibration is a fundamental part of the methods section. Moreover, the presence of the calibration sections in the appendix makes it difficult for the reader to understand the various abbreviations and references that appear later on.

We have now moved some of the calibration steps from the appendix into the main part. But left the material on masks, contact tracing, and summer disease import in the appendix since it has relatively little influence on the overall calibration. We would prefer to leave some of this material in the appendix since otherwise the text to a working model becomes too long.

Additionally, the section that discusses simulation runs should be a part of the methods section.

There is now a section “Time-dependent inputs and calibration”, which mostly contains the calibration material discussed above. The section on simulation runs is the first subsection of that section.

The “Decreasing marginal effect of interventions” and “Intuition for these results” subsections should be a part of the discussion, as they reflect upon the results and do not report them.

We moved “Intuition for these results” into the discussion. We left “decreasing marginal effect of interventions” in the Results section, but anchored it into Table 2, thus clarifying that it *is* a result, and the text is an explanation of that result. We also added a more formal explanation and a figure, see above.
