# Supplementary material for: Predicting the effects of COVID-19 related interventions in urban settings by combining activity-based modelling, agent-based simulation, and mobile phone data
Source: PLoS One. 2021 Oct 28;16(10):e0259037. doi: 10.1371/journal.pone.0259037 (PMC8553173; doi:10.1371/journal.pone.0259037)
Supplement: Attachment — Submitted filename: 2021-09-25 PLOS answer to referees.pdf [file pone.0259037.s011.pdf]

→ NOTE: If you look at the difference pdf, please note that latexdiff has problems with tables, and in consequence the material was modified such that not all differences in tables are shown. The numbers in tables are with one exception the same as before; we extended some headers for clarification. The exception is Tab. 4 where we added the categories of "shop" and "errands", and reduced "home" accordingly.

Reviewer #1: The revision addressed most of my raised points from my review. However, I still see some possible drawbacks of the proposed method, which I would like to be replied to before advising for its publication:

- I like the added section on Out-of-Sample prediction; however, some benchmark comparison to other models (SEIR or mechanistic models, like yesterday is today) would be interesting.

- Why was the RMSLE used for prediction? As far as I know, this is no standard measure. Why not use the RMSE or some proper scoring rule?

Answer to both comments:

We added the following text into the calibration section:

"RMSLE has the advantage that it is less sensitive to the scale of the data than RMSE. That is, relative errors in valleys have as much weight as relative errors on the ridges. This also corresponds to the visual impression of the logarithmic plots often used in epidemics and used throughout the paper. Results using RMSE instead of RMSLE and some more discussion can be found in \nameref{sec:calibr-error-metric}."

The nameref goes to supplementary material S4 Text, which now also contains material for RMSE.

We looked for calibration results by other teams. There is a systematic review paper by Hazelbag at al (https://doi.org/10.1371/journal.pcbi.1007893) which is obviously useful in that direction, but its main thrust (as we understand it) is that models should move from subjective to objective calibration to be policy relevant; we could not find anything on "typical" goodness-of-fit measures for this type of model in that paper.

Chang et al (https://doi.org/10.1038/s41586-020-2923-3) report RMSE of around 400 (see caption of Fig.1), but presumably this needs be seen relative to the scale, which is of the order of 1000 (daily new cases). One might compare this to our own RMSE values, now in S4 Text, which go down to below 100, also on a scale of the order of 1000, but presumably somewhat smaller than the aggregate sample used by Chang et al. This dependency on the scale is also a good argument (we think) for using the logarithmic error (as we do), since that shows the relative error, which is, presumably, what we are interested in (since normally we have, say, hospital beds per capita). Chang et al also calibrate only against the first wave, while picking up the 2nd wave was an important motivation for much material in the present paper.

Aleta et al (http://dx.doi.org/10.1038/s41562-020-0931-9) say on the issue of calibration: “The model is not calibrated to account for the specific evolution of the COVID-19 epidemic in Boston, as it is aimed at showing the effect of different non-pharmaceutical interventions rather than providing a forensic analysis of the outbreak”.

We agree that calibration is important. However, we think that more on calibration would go beyond the scope of this initial paper, which is already long as it stands. Clearly, calibration will have to be addressed if the model gets used more regularly (also because manual calibration takes too much time).

(The data assimilation system of weather forecasting, where one of us comes from, also was a multi-year multi-person effort.)

- Could you add some explanation or comments on two findings of the predictive assessment: Why is the prediction error in the first run under frozen activity levels so high? And it also seems as if updating the activity levels did, in fact, not lead to a better performance in all runs. What does this tell us about the model? Maybe some plots on how the activities changed in that time would be a starting point for the explanation?

This actually was already in the discussion, but evidently at the wrong place. We now moved it to the end of the “Out of sample prediction” section, and adapted it somewhat; it now reads:

“Particularly striking is the blue curve (``2020-05-01''): on the left, the activity participation level is frozen at 71\% (cf.~Table~\ref{tab:out-of-sample-theta}), while on the right it varies mostly between 80\% and 100\% as given by the data plotted in Fig~\ref{fig:reducedParticipation2}. This is consistent with the theoretical argument (Sec.~\nameref{sec:decreasing-effect}) that an activity participation of 71% reduces infections to $71\% \cdot 71\% \approx 50\%$ while an activity participation of 90% reduces infections only to $91\% \cdot 90\% \approx 80\%$. That difference is sufficient to generate the difference between the two curves.”

The plot “on how the activities changed” is actually Fig 9. The difference between the frozen vs. the actual activities calibration is that the slope in fall is somewhat different: In the frozen version, the slope of the model is consistently too low in the fall, but it “crosses” the real data, which leads to smaller RMSLE. In the version with the actual activities, the model output has the right slope, but lies consistently above the real data, leading to slightly larger RMSLE. For the disease management, getting the slope right was important, which may have influenced some other model choices.

- Generally, the size of the Figures should be consistent. Fig. 17 is a lot smaller than, e.g., Fig. 18

done. thanks.

- While I admire the additional work put into extending the paper, I believe that 30 Pages is too long for the main article. I would put some parts of the discussion into the Appendix and most parts of the Appendix into an Online Supplementary Material (if this is possible with Plos One). Maybe also some of the cut discussion from the previous sections "Using mobile device data to observe changes of mobility behavior during COVID-19" and "From reductions of mobility behavior to reductions of infections" could be interesting in the Appendix of the Paper.

We have now fully removed the sections "Location information from mobile device data" and "Using mobile device data to observe changes of mobility behavior during COVID-19" from the manuscript. They were recommended to us by readers for earlier versions of the manuscript, but we agree that they rather detract from the main line of thought.

We have also moved everything that is not strictly necessary for the line of arguments into supplementary material. Beyond what was already there (reduced activity participation by activity type, senozon method, model history), this now concerns "error metric" (i.e. the RMSE results), "robustness runs" (removing the mask model; changing the temperature model), "comparison to other models" and "making the model more realistic". Having the last two items in supplementary material also somewhat shortens the list of references of the main paper.

The main paper now ends on page 26, which indeed seems much reasonable.

We tried to move even more material out of the main part of the paper, but found that it severely affects the flow of the arguments. We also want to point out that the other referee asked us to move some of the calibration material from appendix to main paper to improve the flow of the arguments.

We hope that the referees can agree with these changes; evidently, it would be easy to move material back into the main paper.

- For completeness, a short explanation of the Whitepaper of the Senozon Method in English (the language of the article itself) would be helpful. Besides, some rationale and legitimisation would benefit the article on why a model for transportation is also suitable for modelling COVID hospitalisation

The senozon method is described in S1 Text. We think that this would be sufficient to reconstruct the method if one had the right input data. The exact calibration of the method is not public anyways. We think that this is acceptable since we provide the output of the method, and therefore the input to our model, as open data. Also, the data is used regularly as input to transport simulations.

A sentence on the rationale seems to have gotten lost. We added the following at the beginning of "person-centric epidemiological models derived from transport simulations":

"From the section on person-centric epidemiological modelling above, one takes away that having person trajectories, and in particular where persons meet, would be a good starting point

for an epidemiological simulation. In consequence, the synthetic person trajectories from transport modelling explained above seem like a good starting point, since they are already available."

We also think that our introduction and conclusion make that point:

Introduction: "When COVID-19 took hold in Germany in February 2020, there was an urgent need for a differentiated modelling capability to predict the consequences of interventions. We used our experience with person-centric modelling of traffic \cite{Horni2016-ge} to build a first prototype within two weeks \cite{Muller2020-wv}. An advantage of using this starting point is that the whereabouts of all simulated persons, including their overlapping time spent at facilities or in (public transport) vehicles, are already given by the model, which is derived in part from mobile phone data. Since the input data contains age as an attribute of each synthetic person, it was straightforward to include agent-dependent disease progression into the model from the start. A short description of the different model variants over time is provided in \nameref{sec:modelHistory}."

Conclusion: "We combine a person-centric human mobility model with a mechanical model of infection and a person-centric disease progression model into an epidemiological simulation model. Different from other models, we take the movements of the persons, including the intervening activities where they can interact with other people, directly from data*, which has already been available for transport planning before the pandemics*. ...", where the half-sentence in italics was now added for clarification.

Reviewer #2: I have read the authors reviewed manuscript and responses and find them satisfactory. Therefore, I recommend accepting the manuscript for publication.

Thank you.
